# From generative AI to the brain: five takeaways

**DOI:** 10.3389/fncom.2025.1718778

**Published:** 2025-11-24

**Authors:** Claudius Gros

**Affiliations:** Institute for Theoretical Physics, Goethe University, Frankfurt, Germany

**Keywords:** generative AI, cognitive neuroscience, attention, predictive coding, chain of thought, quantization

## Abstract

The big strides seen in generative AI are not based on somewhat obscure algorithms, but due to clearly defined generative principles. The resulting concrete implementations have proven themselves in large numbers of applications. We suggest that it is imperative to thoroughly investigate which of these generative principles may be operative also in the brain, and hence relevant for cognitive neuroscience. In addition, ML research led to a range of interesting characterizations of neural information processing systems. We discuss five examples, the shortcomings of world modeling, the generation of thought processes, attention, neural scaling laws, and quantization, that illustrate how much neuroscience could potentially learn from ML research.

## 1 Introduction

A multitude of factors contributes to the current rise of generative artificial intelligence (generative AI). Here we focus on two aspects.

- Algorithmic developments can be formulated in many cases in terms of generic generative principles. These generative principles have proven themselves, giving rise to high-performing machine learning architectures. It is an important question whether corresponding principles may operate in the brain.
- In addition to algorithms, insights regarding general working principles and properties of neural-based information processing systems have been attained. Do these apply also to the human brain?

Machine learning (ML) offers a range of conjectures for the workings of our brain, some of which extend or parallel traditional neuroscience frameworks, while others are new. Cognitive neuroscience should accept the challenge and evaluate these conjectures systematically in the context of wet information processing.

A comprehensive overview of potentially relevant cross-relations between ML and the neurosciences is beyond the scope of this perspective. We will focus instead on five key aspects elucidating the importance of paying attention to the concepts that are being developed for generative artificial intelligence. A flurry of new ideas awaits the scrutiny of cognitive neuroscience.

## 2 World modeling is not enough

The two learning principles, “predictive coding” (neuroscience) and “autoregressive language modeling” are both dedicated to the task of building world models, with the former also having active components (Brodski-Guerniero et al., 2017), operating in addition on distinct scales and modalities (Caucheteux et al., 2023).

For large language models (LLMs), autoregressive language modeling takes the form of next-word predictions. However, ML tells us that world-model building alone is insufficient.

The base- or foundation model, viz the result of word prediction training, does contain the knowledge of the world, as present in the training data. But all it can do is to complete a given input word by word. At this stage, key concepts of relevance for the interaction with users, such as “question” and “answer”, are not yet explicitly encoded.

In the early 2020s, a significant step forward was the realization that the otherwise essentially useless base model can be turned into a cognitive powerhouse via a secondary process, denoted “fine tuning” or “human supervised fine-tuning” (HSFT).

- A core fine tuning objective is to teach the system to generate meaningful responses for a given prompt, and not just engage in text completion.
- Next comes fine tuning of style, political correctness, etc.
- Models may be fine tuned further for specific downstream tasks, specializing the otherwise universal LLM to excel, e.g., in accounting.

It seems likely that equivalent processes would occur in our brains. In ML, the two processes are normally separated, viz performed subsequently. In the brain, world model training and fine-tuning via reinforcements are conceivably active at the same time.

**Takeaway:** ML offers a concrete construction plan for a basic cognitive system: Universal unsupervised world modeling followed by supervised fine tuning. To which extent does the brain follow this recipe?

## 3 Generative principles for human thinking

The autonomous generation of thoughts is considered to be the basis of human intelligence. It is hence remarkable that commercial chatbots started to engage in rudimentary “thinking” by the mid-2020s. The algorithm used is denoted “Chain-of-Thought” (CoT) (Zhang et al., 2025), originally a prompting technique (Wei et al., 2022). It is unclear to which extent human thought processes may be understood within the CoT framework, if at all. The same holds for its generalizations, viz “Chain-of-X” (CoX) (Xia et al., 2024), such as Chain-of-Feedback, Chain-of-Instructions, or Chain-of-Histories. In any case, of interest are the underlying generative principles.

- CoT is one of many possible fine-tuning processes, characterized by a specific objective function.
- The system auto-prompts, appending its own thoughts to the user prompt.
- The response is then generated using the combined prompt: (user input) + (chain of thoughts).

Why are responses substantially better when the LLM thinks for a while? A possible explanation is based on the information bottleneck (IB) framework (Tishby and Zaslavsky, 2015). We recall that the token sequence is


$$\begin{array}{c} \text{user input} \end{array}\to \begin{array}{c} \text{CoT} \end{array}\to \begin{array}{c} \text{output} \end{array}$$

The middle part, the thought processes, can be interpreted to act as an information bottleneck for the cognitive processing between input and output (Lei et al., 2025). This principle can be expressed as an information-theoretical min-max optimization.

- The mutual information between the input and CoT is –minimized–.
  This means that the self-generated thoughts should abstract from the specific formulation of the input, retaining only the overall content.
- The mutual information between CoT and the output is –maximized–.
  This because the latent space, namely the thoughts, should be maximally informative with regard to the final output.

The IB view is not just an abstract cookbook. Instead, it has proven itself as a high-performing training algorithm (Lei et al., 2025).

As an alternative to the notion of an information bottleneck, it has been proposed that CoT-type thinking may be seen as an effort to build a composite object, the final response (Zhu et al., 2025). This interpretation allows to leverage state-of-the-art algorithms for diverse object generation, such as GFlowNet (Bengio et al., 2021), which is used widely in synthetic chemistry.

**Takeaway:** The new views of the functionality of thought processes arising within ML research should motivate us to pose one of the most fundamental questions the neurosciences could consider. Could this provide a possible first step toward an understanding of human thinking?

## 4 No attention without self-consistency

A main driver of generative AI is the self-attention mechanism powering the transformer architecture (Vaswani et al., 2017; de Santana Correia and Colombini, 2022). Regarding the brain, we do not touch here the phenomenology of human attention, or the sometimes controversially discussed question how attention should be defined operatively in psychology and in the neurosciences (Hommel et al., 2019; Wu, 2024). Given this caveat, a few comments can be made:

- The details of how attention works on the level of individual neurons are generally not well understood (Moore and Zirnsak, 2017).
- Top-down attention involves the modulation of sensory processing areas by signals generated in higher brain regions. It is generally assumed that these modulatory processes depend only on the specific attention signals, viz without being coupled to the actual process generating the top-down signal in the first place.
- Bottom-up attention is observed when early areas react to salient features in the sensory input stream. Pop-out features are then forwarded with higher intensity (Connor et al., 2004).

Bottom-up attention could be interpreted as a variant of self-attention, albeit with a reduced dynamic range. The latter because lateral saliency detection evolves only slowly in the adult brain (Hopfinger, 2017).

Operatively, there is a key difference between the current view of top-down and ML attention. In the neurosciences, attention processes operating in early brain regions are investigated separately from the question of how the modulating top-down signals are generated via cognitive control (Badre, 2024), e.g., in the context of cholinergic signaling (Parikh and Bangasser, 2020). No such separation is present in machine learning, for a good reason. Components of larger models develop their own neural language when trained separately (Ludueña and Gros, 2013); models need therefore to be trained in their entirety for the individual components to be able to talk to each other.

The context window of a transformer represents past states, which implies that the self-attention mechanism discussed above operates in the time domain, involving hence working-memory aspects (Hintzman, 1984). This connection has been addressed in the context of modern Hopfield networks (Ramsauer et al., 2020; Ororbia and Kelly, 2023).

**Takeaway:** A full understanding of attention needs to include the self-consistency loop between the generation of attention signals and their subsequent processing.

## 5 Neural scaling laws

Neural scaling laws describe how performance and training times scale with model and/or data size (Kaplan et al., 2020; Hoffmann et al., 2022; Michaud et al., 2023).

As an example consider the relative performance of two fully trained models (A and B), which are identical in all aspects, apart from model sizes. In good approximation the relative performance is then (Neumann and Gros, 2022, 2024)


$$\begin{array}{l} \frac{P_{A}}{P_{A}+P_{B}}\;\sim \;\frac{N_{A}}{N_{A}+N_{B}}. \end{array}$$ (1)

where *N*_*A*_ (*N*_*B*_) are the respective numbers of adaptable parameters, and *P*_*A*_ (*P*_*B*_) the corresponding performances. In addition, larger systems need longer to train. For the training compute *C*, viz for the total amount of resources (chips, time, …) needed to train a model, one finds a quadratic scaling relation,


$$C\;\sim \;N^{2}.$$

This leads to an interesting hypothesis regarding putative limitations to the phylogenetic growth of the brain. Assume we have two humans, H1 and H2, the first with a standard brain size, the second with a brain twice as large. If we take 15 years as the time to train standard human brains, H2 would need 2^2^ = 4 times as long, namely 60 years. After 60 years of growing up, H2 would have higher cognitive capabilities than H1, as given by Equation 1. However, ML finds that performance remains somewhat flat during most training, increasing rapidly only at later stages. This implies that H2 would underperform H1 for extended periods, say the first 40–50 years of adolescence. Evolutionary speaking, doubling brain size may hence not be a viable option. Of course other limiting factors, like metabolic costs, may have determined the size of our brains.

**Takeaway:** Given that information processing networks characterize not only modern machine learning architectures, but also the brain, the biological implications of neural scaling deserve to be investigated.

## 6 Quantization

Large models have large numbers of adaptable parameters, which one needs not only to store, but also to keep in working memory, ready for subsequent use. Typical floating point datatypes are 32 bits (or 64 bits for double precision). In order to save memory, and to make operations faster, data sizes have been reduced in recent years (Wei et al., 2024; Gong et al., 2025). Currently, INT4 (4 bits) devices are being rolled out. For INT4, one has just 2^4^ = 16 possible values. Synaptic weights are hence “quantized”, taking only one out of 16 possible states. Specialized GPUs support the involved operations efficiently. In analogy, synaptic strength is quantized also in the brain (Petersen et al., 1998; Liu et al., 2017), with the exact number of expressed states being debated.

**Takeaway:** The computational consequences of synapse quantization are well understood for artificial neural nets. This knowledge should be readily transferable to their biological counterparts.

## 7 Conclusions

We reviewed five selected concepts contributing to the rapid progress of generative AI. Interestingly, in machine learning literature, their relevance to biological information processing systems is rarely discussed, if at all, with attention being in part an exception (Lindsay, 2020). The scope of this perspective is to raise awareness that a treasure of generative principles may be hidden in ML literature.

## Data Availability

The original contributions presented in the study are included in the article/supplementary material, further inquiries can be directed to the corresponding author.
